# Distribution of Intranasally Administered rIL‐10 Along the Olfactory Nerve and Perivascular Space After Intracerebral Hemorrhage

**DOI:** 10.1111/cns.70372

**Published:** 2025-04-16

**Authors:** Shaoshuai Wang, Junmin Wang, Xinru Zhang, Shijun Xu, Qinfeng Peng, Yifei Li, Ruoqi Ding, Bing Jiang, Shuyu Wang, Shuaibing Zhang, Siyuan Hu, Yousef Rastegar‐Kashkooli, Na Xing, Nan Li, Menglu Wang, Junyang Wang, Xuemei Chen, Chao Jiang, Xiaochong Fan, Jian Wang

**Affiliations:** ^1^ Department of Pain Medicine The First Affiliated Hospital of Zhengzhou University Zhengzhou China; ^2^ Department of Human Anatomy, School of Basic Medical Sciences Zhengzhou University Zhengzhou China; ^3^ Non‐Commissioned Officer School of Army Medical University Shijiazhuang China; ^4^ School of Pharmacy Chengdu University of Traditional Chinese Medicine Chengdu China; ^5^ Institute of Material Medica Integration and Transformation for Brain Disorders, Chengdu University of Traditional Chinese Medicine Chengdu China; ^6^ Nanozyme Laboratory in Zhongyuan, School of Basic Medical Sciences Zhengzhou University Zhengzhou China; ^7^ Nanozyme Laboratory in Zhongyuan Henan Academy of Innovations in Medical Science Zhengzhou China; ^8^ Department of Anesthesiology, Pain and Perioperative Medicine The First Affiliated Hospital of Zhengzhou University Zhengzhou China; ^9^ Department of Neurology The Second Affiliated Hospital of Zhengzhou University Zhengzhou China; ^10^ Department of Biochemistry and Molecular Biology, School of Basic Medical Sciences Zhengzhou University Zhengzhou China; ^11^ Department of Neurology People's Hospital of Zhengzhou University and Henan Provincial People's Hospital Zhengzhou China

**Keywords:** BBB, ICH, in vivo imaging, intranasal administration, rIL‐10

## Abstract

**Rationale:**

The utilization of anti‐inflammatory therapy for treating brain diseases holds promise; however, research on intranasal administration of drug compounds remains limited. Quantitative data, particularly pharmacokinetics, are scant, and direct evidence of the distribution of intranasally administered recombinant interleukin 10 (rIL‐10) within the brain is lacking.

**Methods:**

Employing fluorescent labeling, in vivo imaging, and confocal microscopy, we meticulously monitored the distribution and delivery pathways of intranasally administered rIL‐10 in the brain.

**Results and Conclusions:**

Our findings demonstrate that rIL‐10 can permeate the blood–brain barrier and reach the perihematomal area in the striatum of mice with intracerebral hemorrhage. Intranasally administered rIL‐10 primarily targets the cerebral cortex, striatum, and thalamus, traversing the olfactory nerve pathway and perivascular space to access these brain regions. This mode of delivery effectively mitigated secondary brain injury after intracerebral hemorrhage. This study contributes to intranasal drug delivery research, offering compelling evidence to support the intranasal delivery of anti‐inflammatory cytokines or alternative drug candidates for treating brain diseases.

## Graphical abstract

Delivering exogenous recombinant interleukin‐10 (rIL‐10) via intranasal administration involves dispersing rIL‐10 throughout the brain using the olfactory nerve pathway and perivascular space.

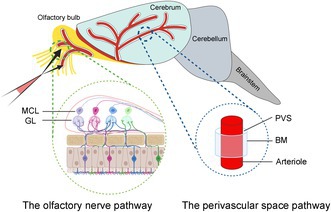

**Abbreviations**
BBB: blood–brain barrier
ICH: intracerebral hemorrhage
rIL‐10: recombinant interleukin 10
rIL‐10‐CY5.5: CY5.5‐labeled recombinant interleukin‐10

## 1 Introduction

There is mounting evidence indicating the involvement of neuroinflammation in a diverse range of neurological disorders, including neurodegenerative diseases and acquired brain injuries [1]. Cytokine therapy, considered a promising treatment for nervous system diseases, has attracted significant attention [2]. The potential of cytokine therapy is immense, offering hope in treating nervous system diseases. However, the therapeutic effectiveness of these medications relies on their ability to penetrate the blood–brain barrier (BBB), a formidable obstacle that restricts the passage of most macromolecules (> 400–600 Da) into the brain tissue. While BBB disruption occurs following intracerebral hemorrhage (ICH), its hyperpermeability is not evident until Day 3 in the animal model [3] and takes 12 h for patients [4] This temporal limitation poses a substantial challenge for early‐stage therapeutic intervention using conventional drug delivery methods, such as intravenous administration. It impedes the targeted delivery of drugs to the brain [5].

Intranasal drug delivery, a noninvasive method, offers a promising solution for bypassing the BBB. It enables the rapid transportation of large molecules to the brain while minimizing systemic exposure [6]. This approach shows promise as an effective intervention for neurological conditions such as Alzheimer's, Parkinson's, and stroke [7, 8, 9]. While preclinical studies using the intranasal route have generally shown positive results, quantitative data on brain delivery, particularly pharmacokinetics, are limited primarily to research on oxytocin [10], nanoparticles [11], and adeno‐associated viral vectors [12]. However, the lack of direct evidence of cytokine delivery to the brain through intranasal administration and the incomplete understanding of its distribution within the brain highlight the need for further research.

Cytokines are critical in safeguarding the brain [13]. Recent investigations have revealed that the intranasal administration of interleukin 10 (IL‐10) has the potential to alleviate depressive behavior and cognitive function in mouse models of depression [14, 15]. Furthermore, intranasal delivery of PSL‐IL‐10 has improved outcomes in ICH [16]. Our prior research has established the ability of recombinant IL‐10 (rIL‐10) to expedite hematoma clearance post‐ICH and facilitate brain repair after traumatic brain injury [17, 18, 19, 20]. Nonetheless, comprehensive education on rIL‐10 distribution within the brain and its intranasal delivery route still needs to be completed. To address this knowledge gap, we utilized fluorescence labeling, in vivo imaging, and confocal microscopy to investigate the rIL‐10 distribution and delivery route in the brain after intranasal drug delivery. This research endeavors to propel the field of nose‐to‐brain drug delivery and accelerate the clinical translation of rIL‐10. Additionally, our research findings will provide valuable insights and serve as a reference for treating brain diseases through intranasal delivery of other drug compounds.

## 2 Materials and Methods

### 2.1 Animals

C57BL/6 male mice that were 8–10 weeks old and weighed 23–25 g SCXK (Beijing) 2019‐0010 were used in the study. The mice were maintained in a specific pathogen‐free (SPF) environment with a 12‐h day‐night cycle, a controlled temperature of 25°C, and a relative humidity of 45%–55%. They had ad libitum access to standard food and water. The experimental protocol for this study was approved by the Animal Ethics Committee of Zhengzhou University (ZZUIRB 2022‐31). All mice were assigned to groups using a randomization website (http://www.randomization.com).

### 2.2 The ICH Model

The procedure for inducing the ICH model was implemented as outlined in our previous publications [18, 21]. Initially, mice were anesthetized using 3.5% isoflurane (4% for induction and 2% for maintenance). Following this, collagenase VII‐S (C2399, 0.05 U in 0.5 μL sterile saline, Sigma, St. Louis, MO, USA) was administered into the striatum of mice to induce ICH at coordinates of 0.8 mm anterior, 2.1 mm lateral, and at a depth of 3.3 mm. Sham surgeries involved isoflurane anesthesia and an identical head skin incision. A warming blanket was employed throughout the surgical process to maintain a constant body temperature. Postsurgery, the mice were housed in a specific pathogen‐free environment with unrestricted access to water and food until complete recovery.

### 2.3 Tissue Harvesting

Following deep anesthesia with isoflurane, mice underwent perfusion with saline through the left ventricle until the liver appeared pallid. Subsequently, perfusion with 4% paraformaldehyde was carried out. The brain tissue was then extracted and immersed in paraformaldehyde for 12 h. The brain was immersed in 20% and 30% sucrose solutions for 24 h each, proceeding with the embedding process in OCT. Brain slices were subsequently obtained at a thickness of 25 μm using a Cryostat.

### 2.4 The Procedure for Binding the Fluorescent Tag CY5.5 to the rIL‐10

Micro‐CY5.5 [PA15605, Cytiva (GE Life)] was added to a centrifugal tube containing 1 mL of DMSO and agitated until fully dissolved. Subsequently, 1 mL of rIL‐10 (417‐ML, R&D Systems) was introduced to the solution and stirred at 4°C overnight. The removal of unbound CY5.5 was accomplished through centrifugation using an ultrafiltration centrifuge tube with a molecular weight cutoff of 3 kDa. Subsequently, 2 μL of rIL‐10 and rIL‐10‐CY5.5 solutions were analyzed using a UV spectrophotometer to assess the labeling success, and the respective peak absorbances were compared. The CY5.5‐labeled rIL‐10 was then aliquoted and stored at −80°C in the dark.

### 2.5 The Protocol for Administering rIL‐10

Pre‐intranasal administration adaptation training was conducted using Hanson's method, followed by intranasal administration in awake mice [22]. The optimal therapeutic dose, as determined by our prior study [18], was administered intranasally. Mice received the initial dose 2 h after ICH, and subsequent twice‐daily dosing was continued until tissue collection.

### 2.6 In Vivo Imaging

Mice were anesthetized with isoflurane and received rIL‐10‐CY5.5 intranasally. Fluorescence imaging was conducted using a small animal in vivo imaging system at specified time intervals postadministration: 15 min, 1 h, 4 h, 12 h, 24 h, 36 h, and 48 h. After imaging, mice were perfused and fixed with paraformaldehyde, followed by brain dehydration using a gradient process. Brain slices, 1 mm thick, were imaged using the same system. Examination for fluorescence was conducted on intact organs (heart, liver, spleen, lung, kidney, and brain). The acquired images were processed and analyzed utilizing the Living Image software (version 4.7.4). Signal strength within the images is denoted by color intensity, with yellow representing high signal and red representing low signal. The data analysis was perforemd in a blind manner.

### 2.7 Sudan Black B (SBB) Treatment

According to our established protocol [23], tissue sections were incubated in a 1.5% SBB solution (199664‐25G, Sigma‐Aldrich) for 5 min, followed by sequential rinses in 70% ethanol (30 s) and phosphate‐buffered saline (5 min).

### 2.8 Neurologic and Motor Function Evaluations

The mice were randomly assigned to treatment groups using numbers from a randomization number generator website, ensuring an unbiased distribution across the experimental conditions. On D3 post‐ICH, an investigator unaware of the treatment groups assessed the mouse neurologic deficit score and motor function. Neurological deficit scores were assessed following ICH at specified time intervals, as detailed in previous literature [18]. Behavioral tests, including body symmetry, gait, climbing, circle‐twisting behavior, forelimb symmetry, and forced circle‐twisting, were performed in a blind manner. Each test was scored on a scale from 0 to 4, with the cumulative scores documented for individual subjects. The wire‐hanging test involved placing mice on a horizontal wire, and the suspension duration was recorded and averaged across three trials, ensuring the accuracy and reliability of the results. Locomotor and coordination impairments were evaluated using the inverted grid test [24]. Each animal was positioned in the center of an inverted wire mesh, and the suspension duration was monitored until the mouse fell or remained motionless for 3 min. This procedure was repeated thrice for each subject, and the average of these trials was considered the final measurement. Sponge pads were strategically positioned beneath the mesh to mitigate the risk of falls.

### 2.9 CV/LFB

As previously reported [21], the mouse brain tissue underwent histologic analysis involving embedding, sectioning, and staining with Crystal Violet (CV, C5042, Sigma‐Aldrich) and Luxol fast blue (LFB, S3382, Sigma‐Aldrich). The stained brain sections were acquired microscopically, and image analysis was performed using the ImageJ software. Adhering to established protocols, the lesion volume was determined by summing the lesion area and the interlayer spacing. We conducted blind data analysis to reduce the risk of potential bias.

### 2.10 Fluoro‐Jade B (FJB) Histology

In the process of FJB staining, brain sections were subjected to the following sequential treatments: a 5 min exposure to a 1% sodium hydroxide 80% ethanol solution, a 2 min decolorization stage using a 70% ethanol solution, and a final rinse with double‐distilled water. Subsequently, the sections were subjected to a 20 min incubation in a 0.06% potassium permanganate solution on a shaking platform. The brain sections were then immersed in a 0.0004% FJB solution (USA, Biosensis, BSS‐TR‐150‐FJB) and incubated in darkness for 20 min. The stained sections were then thoroughly rinsed with double‐distilled water for 3 min, dried at 60°C, and preserved by mounting them in neutral resin. A representative sample comprising three brain sections from each mouse was randomly selected for subsequent statistical analysis. Each section was photographed in four distinct regions surrounding the hematoma: upper, lower, left, and right. This process yielded 12 images for each mouse, subsequently averaged for statistical analysis. The acquired images were analyzed using the ImageJ software, and the blind data analysis was performed to ensure objectivity.

### 2.11 Perls' Blue Staining

Iron deposition was detected using Perls' blue staining kit (G1420, Solarbio) following a previously published protocol [18]. Briefly, the working solution was applied to the brain slice and incubated at 37°C for 20 min. After three rinses with distilled water, the incubation working solution was reapplied to the brain slice and incubated again at 37°C for another 20 min. Finally, the brain slice was washed three times with PBS. The enhancement working solution was applied to the brain slices and incubated at 37°C for 10 min, followed by three washes with PBS. Subsequently, the restaining solution was added and incubated for 5 min, after which the brain slices were washed with distilled water for 10 min. Three brain slices were randomly selected from each mouse, and each slice was photographed in four different fields of view surrounding the hematoma. The average number of iron‐depositing cells in the 12 fields was then analyzed using the ImageJ software, which remained blind throughout the analysis.

### 2.12 Immunofluorescence

Immunofluorescence staining was conducted on brain sections obtained after ICH according to a previously published protocol [23]. The frozen brain sections were first blocked with 1% Triton X‐100 and 5% sheep serum for 2 h. Next, the anti‐rabbit Iba‐1 antibody (USA, Affinity, DF6442) or anti‐mouse endothelial cell antigen‐1 (USA, Abcam, ab9774) was applied and incubated overnight at 4°C. After incubation, the sections were washed thrice with PBS for 10 min each. Subsequently, goat anti‐rabbit antibody (USA, Jackson, JAC‐111‐545‐003) or goat anti‐mouse antibody (USA, Abcam, ab150113) was incubated for 2 h at room temperature, followed by three washes with PBS. The slices were stained with DAPI for 1–2 min, washed thrice with PBS, and finally sealed with an anti‐fluorescence quencher. Three brain slices were randomly selected from each mouse and photographed in the four fields surrounding the hematoma: upper, lower, left, and right. Photographs were captured and imaged in three‐dimensional images using a confocal microscope (Leica, THUNDER Imaging Systems). Twelve fields of view were captured from each mouse for quantitative analysis. After ICH, activated microglia/macrophages were identified as Iba‐1‐positive cells. These activated cells displayed spherical, amoeboid, or rod‐shaped morphology, with cell body diameters exceeding 7.5 μm in at least one dimension and featuring short, thick processes [25]. The number of these activated Iba‐1‐positive cells surrounding the lesion was analyzed and counted using ImageJ. The statistical process was conducted in a blind manner.

### 2.13 Statistical Analysis

The statistical analysis and mapping of experimental data were conducted using GraphPad Prism 8.4.3 (GraphPad Software; Inc., La Jolla, CA). The experimental data were expressed as mean ± standard deviation (means ± SD). The normal distribution of the results was confirmed using the Shapiro–Wilk test. A *t‐test* statistical analysis was employed to compare data from the two groups. In contrast, an analysis of variance (ANOVA) followed by an appropriate *post hoc* test was used to compare data among multiple groups, and *p* < 0.05 was considered statistically significant.

## 3 Results

### 3.1 Pharmacokinetic Analysis of Intranasal Administration of rIL‐10‐CY5.5 in the Brain

**FIGURE 1:**
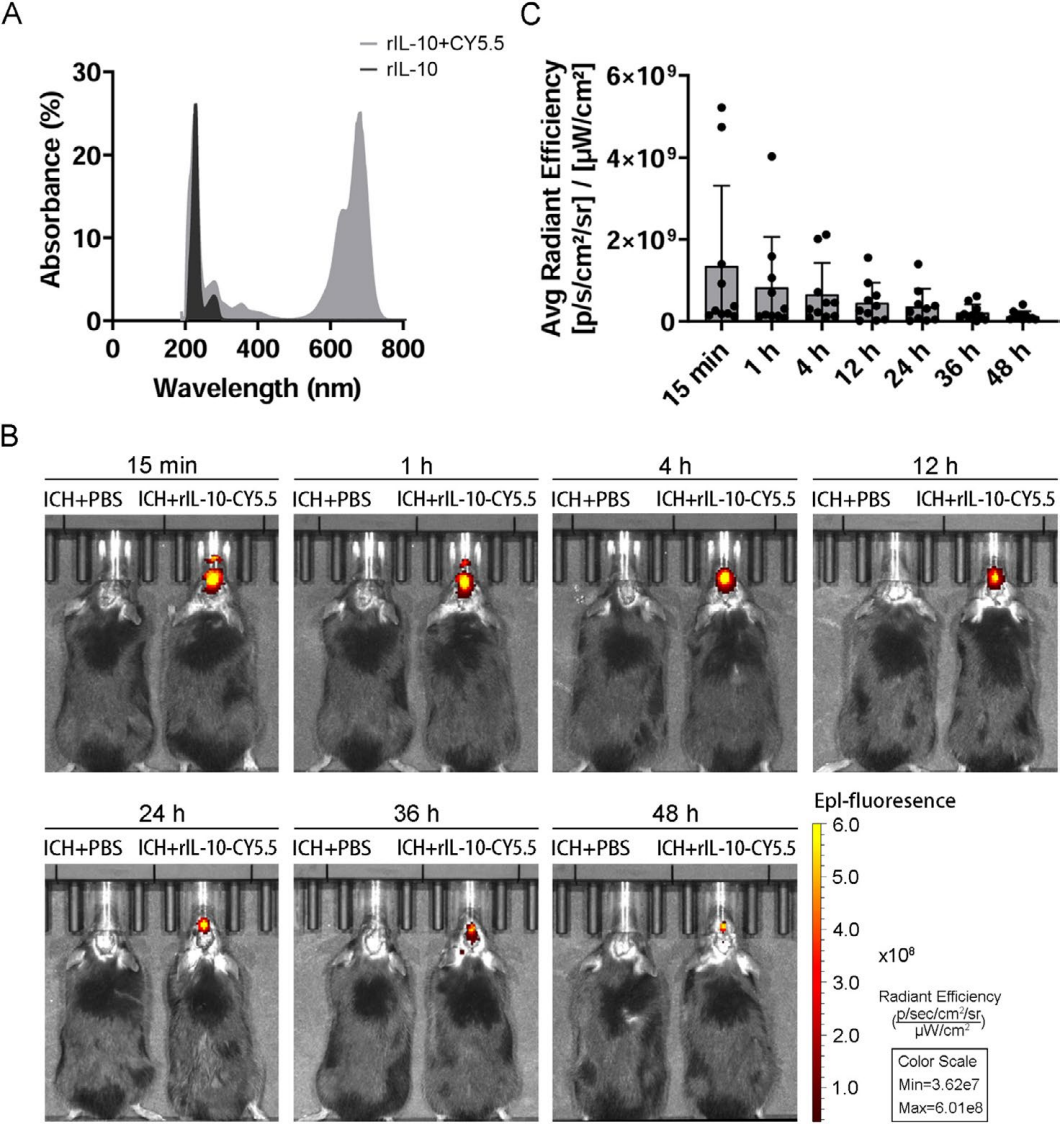
Dynamics of intranasal administration of exogenous CY5.5‐labeled recombinant interleukin‐10 (rIL‐10‐CY5.5) in the intracerebral hemorrhage (ICH) model. (A) Representative UV–vis–NIR spectrum of rIL‐10‐CY5.5. rIL‐10‐labeled CY5.5 demonstrated a typical peak compared to rIL‐10, with excitation wavelengths consistent with those of CY5.5. (B) In vivo images of intranasal administration of rIL‐10‐CY5.5 at different time points in the same mouse. (C) Statistics of average fluorescence intensity in brain regions of mice imaged in vivo at different time points (*n* = 10).

To investigate the distribution of intranasally administered rIL‐10 within the brain, rIL‐10 was conjugated with a CY5.5 fluorescent label. The absorbance of the conjugate was subsequently measured using UV spectrophotometry, revealing a distinct absorption peak at 673 nm for rIL‐10‐CY5.5, thus confirming the successful coupling of rIL‐10 with the CY5.5 fluorescent label (Figure 1A). In vivo imaging technology was then used to monitor the fluorescence intensity and distribution at various time points following a single intranasal administration, demonstrating a pronounced fluorescent signal in the nasal cavity and nearby olfactory bulb of the brain in mice 15 min post intranasal administration of rIL‐10‐CY5.5, in comparison with the control group. Subsequently, the fluorescent signals in the nasal cavity gradually migrated toward the brain and were fully relocated to the brain after 4 h. Eventually, these signals spread to the other brain regions and concentrated within the whole brain (Figure 1B). After that, the fluorescence signal gradually diminished over time, fading entirely after 48 h (Figure 1C).

### 3.2 The Distribution of rIL‐10‐CY5.5 Following Intranasal Administration in Major Organs

**FIGURE 2:**
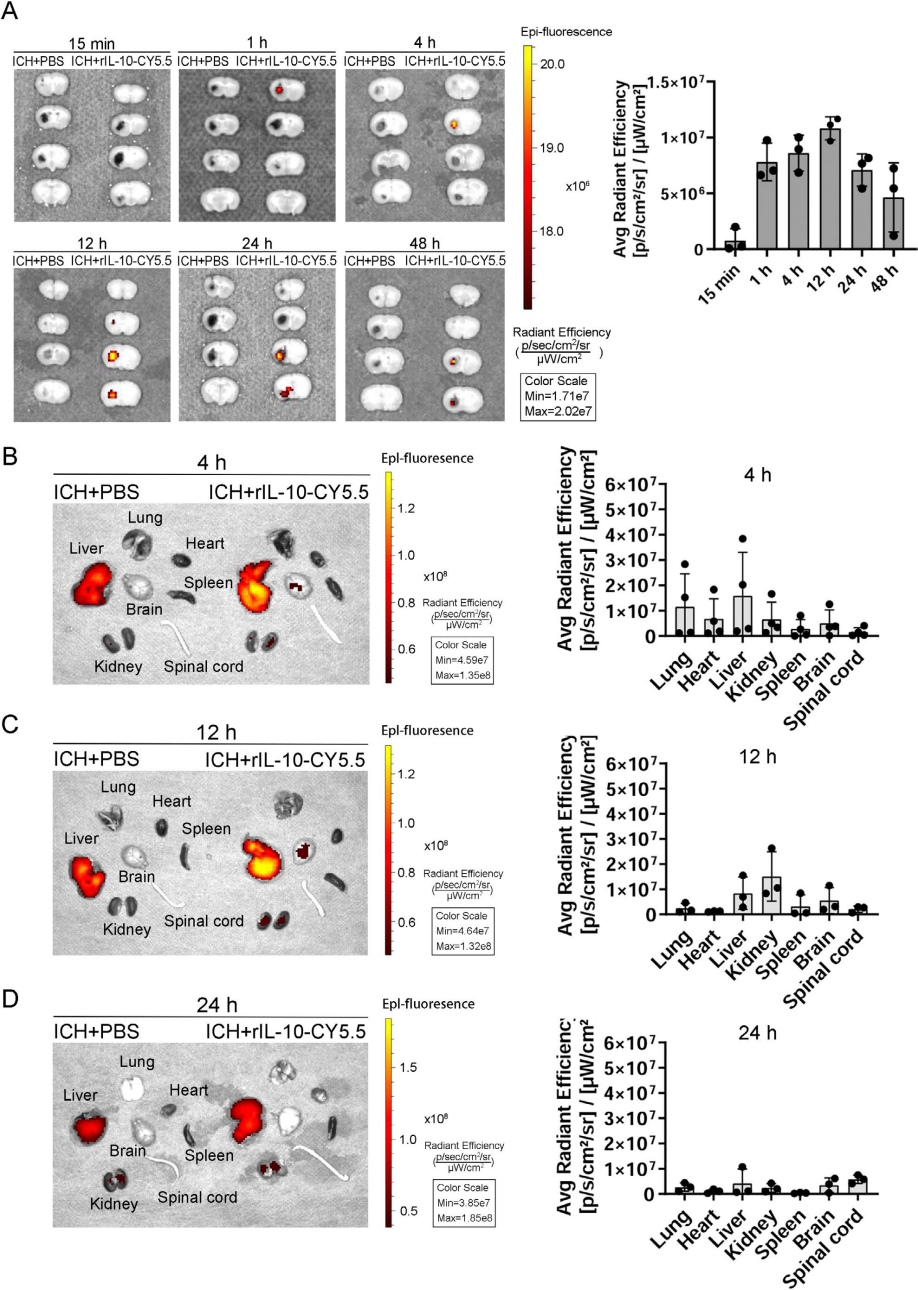
Fluorescence distribution in the main organs after the intranasal administration of exogenous CY5.5‐labeled recombinant interleukin‐10 (rIL‐10‐CY5.5) in intracerebral hemorrhage (ICH) model. (A) Distribution and quantitative statistics of fluorescence signals in ex vivo brain: Fresh brain slices were collected at 15 min, 1 h, 4 h, 12 h, 24 h, and 48 h after intranasal administration of exogenous rIL‐10‐CY5.5. These slices were then subjected to fluorescence imaging and data analysis. *n* = 3. (B) Distribution and quantitative statistics of fluorescence signals in different ex vivo organs at 4 h after intranasal administration of exogenous rIL‐10‐CY5.5. *n* = 4. (C) Distribution of fluorescence signals in different ex vivo organs at 12 h after intranasal administration of rIL‐10‐CY5.5. *n* = 3. (D) Distribution and quantitative statistics of fluorescence signals in different ex vivo organs at 24 h after intranasal administration of rIL‐10‐CY5.5. *n* = 3. We used ICH mice treated with PBS as controls.

The rIL‐10 distribution following intranasal administration was investigated in major organs through mouse brain dissection and subsequent organ harvesting at various time points. Fluorescence imaging of fresh brain sections in vitro revealed the initial appearance of the fluorescence signal near the anterior cerebrum at 1 h, with subsequent concentration primarily around the hematoma 12 h before gradual diminishment at 24 h (Figure 2A). Additionally, we found no difference in rIL‐10 fluorescence signals between the hematoma side and the contralateral side at different time points (Figure S1). Imaging results from other organs indicated predominant distribution in the lung, heart, liver, and kidney 4 h post intranasal administration (Figure 2B). Subsequently, at 12 h post administration, rIL‐10 was notably concentrated in the liver, kidney, and brain (Figure 2C), and at 24 h, distribution was observed in the brain and spinal cord (Figure 2D).

### 3.3 Intranasal Administration of rIL‐10‐CY5.5 Is Transported to the Brain Through the Olfactory Nerve and Perivascular Space

Intense autofluorescence occurs in and around the hematoma after ICH. Our research team has developed a protocol to reduce autofluorescence effectively using SBB treatment [23]. In this experiment, we used SBB to eliminate autofluorescence before conducting fluorescence imaging. This process is illustrated in Figure S2.

**FIGURE 3:**
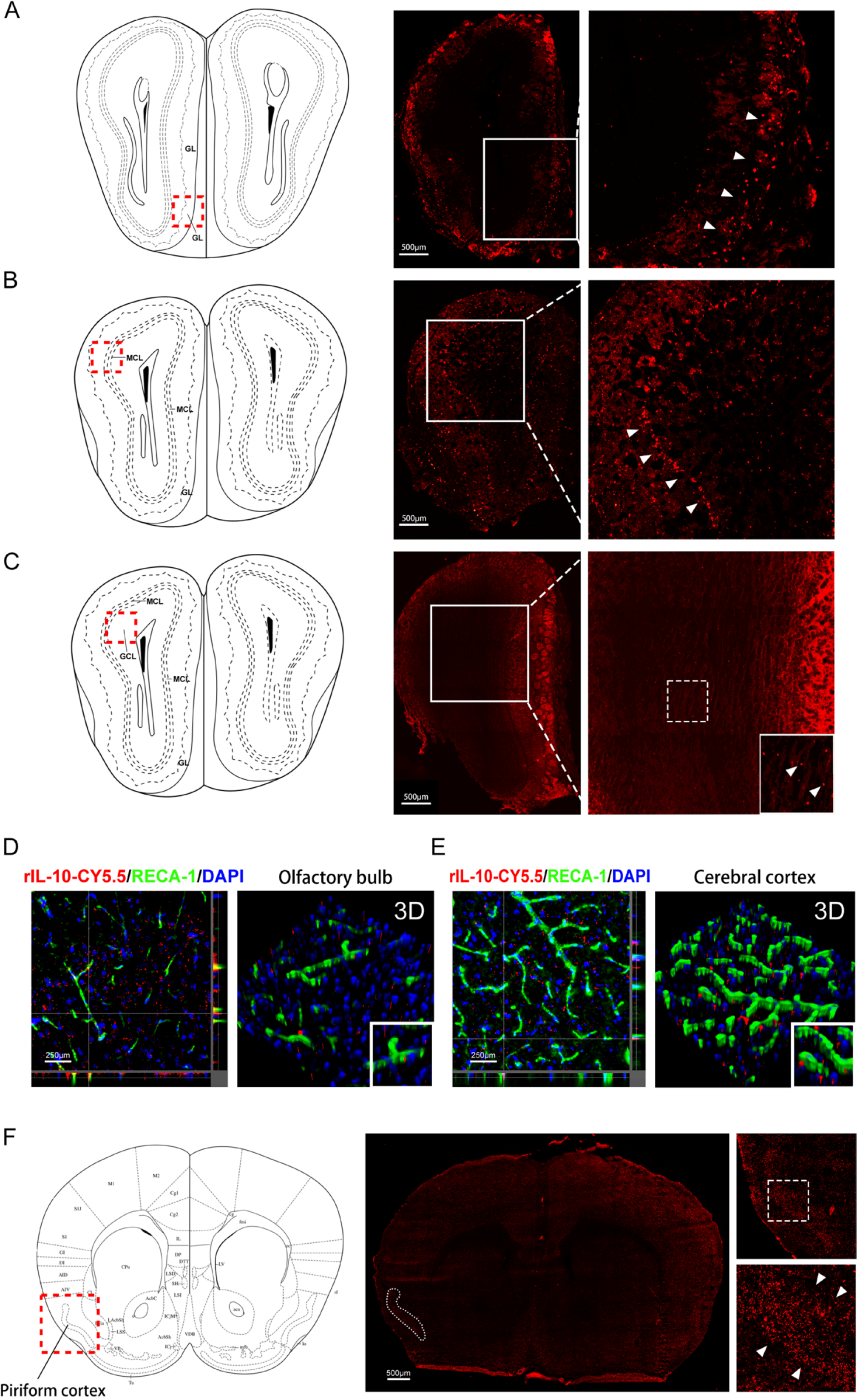
Delivery route for intranasal administration of CY5.5‐labeled recombinant interleukin‐10 (rIL‐10‐CY5.5). Mouse olfactory bulb sections were collected at 1 h after intranasal administration of rIL‐10‐CY5.5. (A) rIL‐10‐CY5.5 fluorescent distribution was observed in the olfactory bulb's glomerular layer (GL) at 8.08 mm interaural and 4.28 mm bregma. (B) rIL‐10‐CY5.5 fluorescent distribution was observed in the olfactory bulb's mitral cell layer (MCL) at 7.72 mm interaural and 3.93 mm bregma. (C) Fluorescence distribution in the olfactory bulb's granule cell layer (GCL) at 7.72 mm interaural and 3.93 mm bregma. (D) Confocal microscopy was used for immunofluorescence imaging on brain slices from the olfactory bulb granule cell layer at 2 h after intranasal administration. rIL‐10‐CY5.5 (red), blood vessels (RECA‐1, green), nucleus (DAPI, blue). The images were captured in 3D. (E) Confocal microscopy was used for immunofluorescence imaging on cerebral cortex brain slices at 2 h after intranasal administration. (F) Distribution of fluorescence signal was observed in the piriform cortex of the mouse brain (Interaural 5.14 mm, Bregma 1.34 mm). The white arrows indicate representative fluorescence signals.

We used fluorescence imaging to track rIL‐10‐CY5.5 dissemination from the nose to the brain. Two hours later, we detected high levels of fluorescence in the glomerular layer (GL) and mitral cell layer (MCL) of the olfactory bulb, as well as in the pyriform cortex (Figure 3A,B,F). This anatomical distribution of the signal suggests that rIL‐10‐CY5.5 traveled from the olfactory bulb to the brain along the olfactory nerves. High fluorescence signals were also found in the granular cell layer (GCL) and cerebral cortex (Figure 3C). To understand how rIL‐10‐CY5.5 was delivered and distributed within the GCL and cerebral cortex, we used confocal and 3D imaging with RECA‐1‐labeled blood vessels. These techniques revealed that rIL‐10‐CY5.5 fluorescence was localized within the perivascular space (PVS) (Figure 3D,E). Our findings suggest that rIL‐10 primarily travels along the olfactory nerve and spreads along the perivascular space.

### 3.4 The Distribution of rIL‐10‐CY5.5 in Various Brain Regions

**FIGURE 4:**
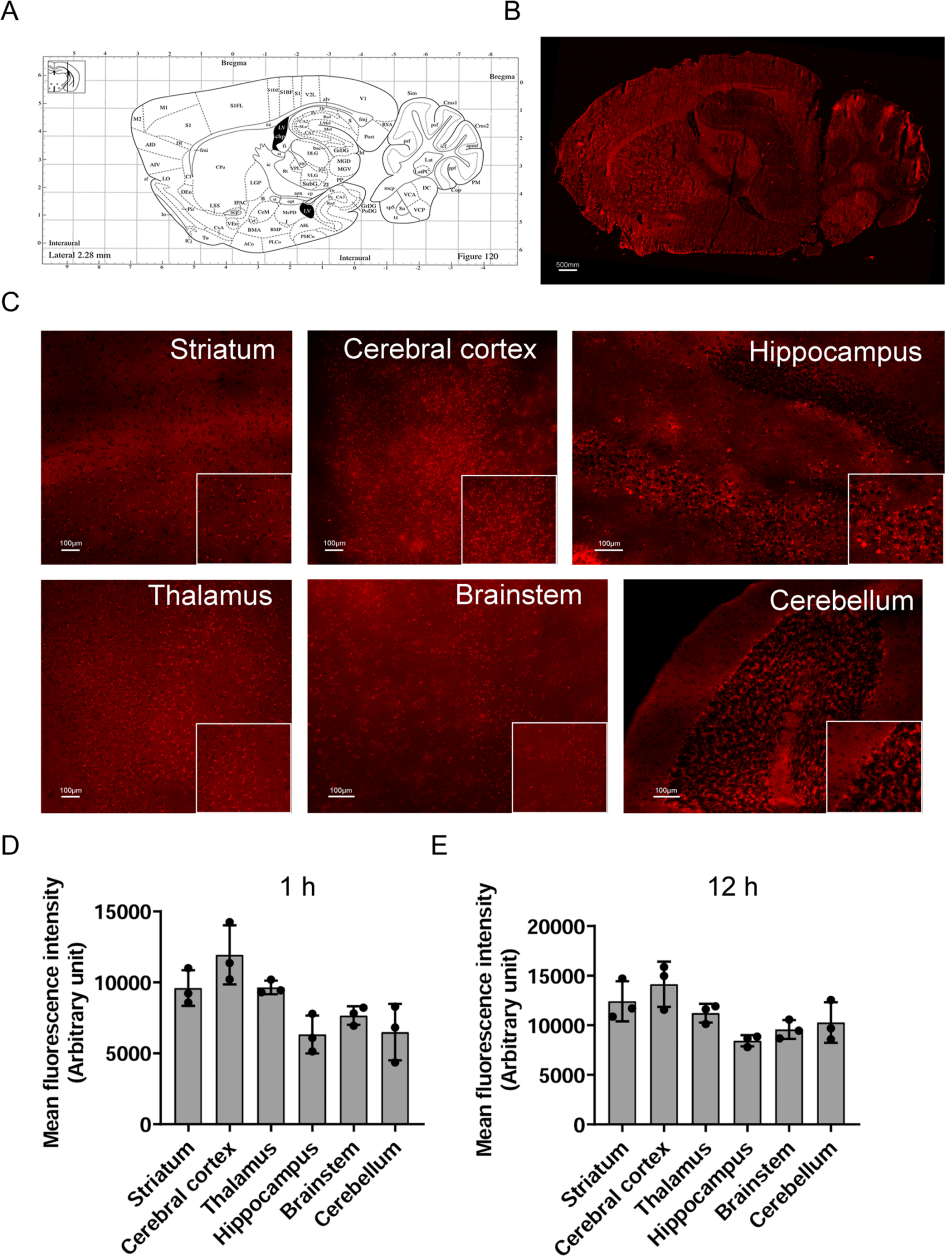
Fluorescence distribution of CY5.5‐labeled recombinant interleukin‐10 (rIL‐10‐CY5.5) in different brain regions of mice after intranasal administration. (A) Schematic diagram of a sagittal section of the mouse brain, corresponding to the position of the brain slices in B. (B) Fluorescence distribution of rIL‐10‐CY5.5 in sagittal brain sections at 1 h after intranasal administration. (C) Fluorescence distribution of rIL‐10‐CY5.5 in different brain regions at 1 h after intranasal administration. (D) Statistics of fluorescence intensity in different brain regions of mice at 1 h after intranasal administration of rIL‐10‐CY5.5. (E) Statistics of fluorescence intensity in different brain regions of mice at 12 h after intranasal administration of rIL‐10‐CY5.5. *n* = 3 mice/group.

We examined how rIL‐10 spreads to different brain areas after intranasal administration by analyzing the fluorescence distribution pattern in the sagittal section of the brain (Figure 4A,B). Additionally, we measured the fluorescence intensity at 1 h in the striatum, cerebral cortex, hippocampus, thalamus, brainstem, and cerebellum (Figure 4C). Our findings revealed that the cerebral cortex and thalamus exhibited the highest fluorescence levels 1 h after intranasal administration. In contrast, the hippocampus and cerebellum showed the lowest levels (Figure 4D). Furthermore, after 12 h of intranasal administration, the striatum and cerebral cortex displayed the highest fluorescence levels, while the hippocampus and brainstem exhibited the lowest levels (Figure 4E). The presence or absence of hematoma did not influence the rIL‐10 distribution across various brain regions, including the striatum, cerebral cortex, thalamus, hippocampus, brainstem, and cerebellum (Figure S3).

### 3.5 Intranasal Administration of rIL‐10 Mitigates Secondary Brain Injury After ICH

**FIGURE 5:**
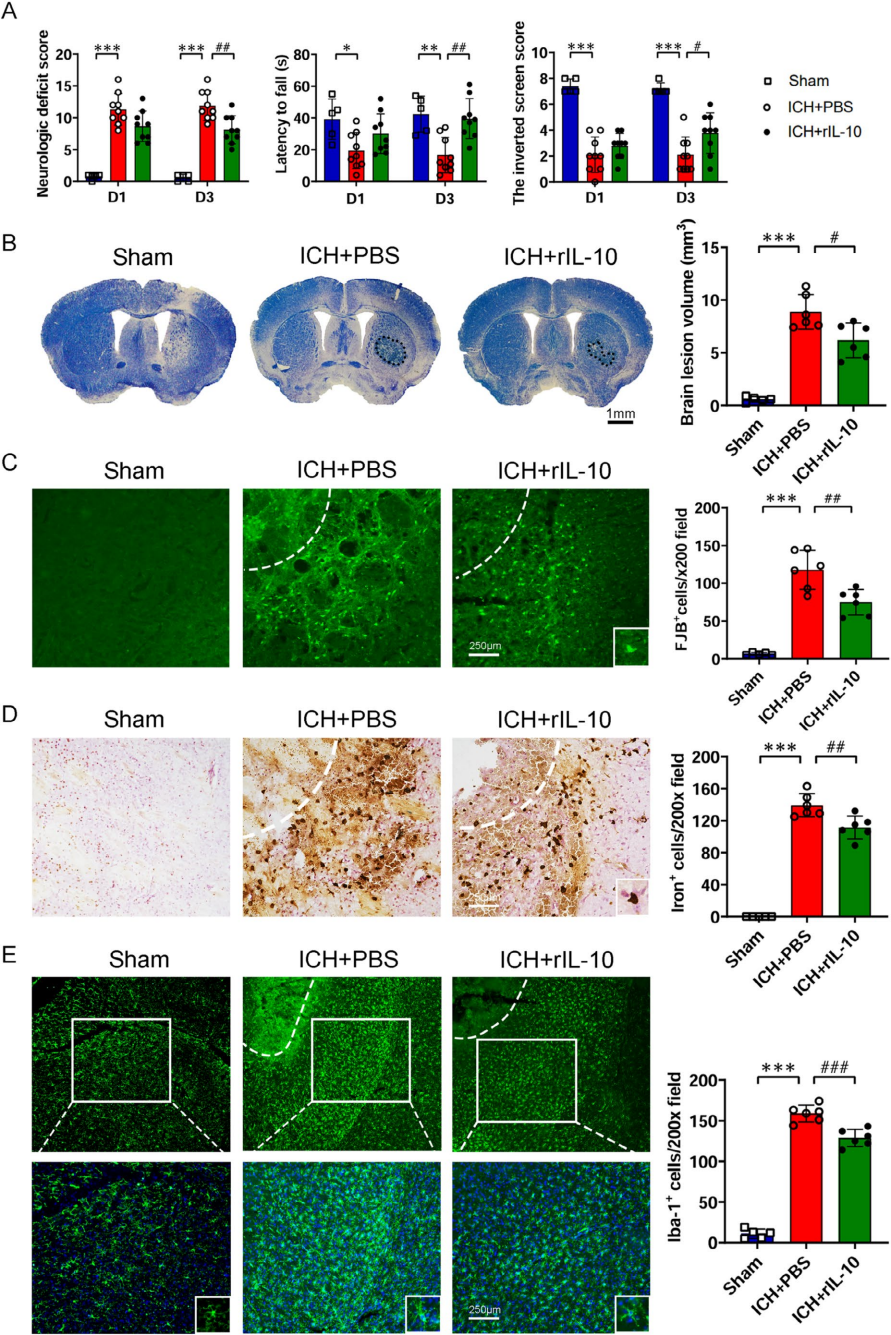
Intranasal administration of recombinant interleukin‐10 (rIL‐10) improved neurologic function after intracerebral hemorrhage (ICH). (A) Intranasal administration of rIL‐10 after ICH improved neurologic function on Day 3. The experimental groups included the sham group: *N* = 5, the ICH + PBS group, and the ICH + rIL‐10 group; *n* = 9. *p < 0.05, **p < 0.01, ***p < 0.001 versus corresponding sham group; ^#^ *p* < 0.05, ^##^ *p* < 0.01 versus the ICH + PBS group. Neurologic deficit score, *F*_Interaction_ = 0.3177, *F*_Row Factor_ = 0.01054, *F*_Column Factor_ = 85.7; Wire hanging test, *F*_Interaction_ = 1.186, *F*_Row Factor_ = 0.8134, *F*_Column Factor_ = 14.63; Reverse grid test, *F*_Interaction_ = 1.09, *F*_Row Factor_ = 0.5148, *F*_Column Factor_ = 60.54; Two‐way ANOVA followed by Sidak multiple comparison post hoc test was used for analysis. (B) The lesion volume was calculated by Cresyl Violet/Luxor fast blue (CV/LFB) staining on Day 3 post‐ICH. *n* = 5–6. ***p < 0.001 versus sham; ^#^ *p* < 0.05 versus ICH + PBS. One‐way ANOVA followed by Tukey multiple comparison *post hoc* test was used for statistical analysis. (C) Degenerating neurons were calculated by Fluoro‐Jade B (FJB) staining on Day 3 post‐ICH. *n* = 5–6. ***p < 0.001 versus sham; ^##^ *p* < 0.01 versus ICH + PBS. One‐way ANOVA followed by Tukey multiple comparison *post hoc* test was used for statistical analysis. (D) Iron‐positive cells were calculated by Perls' staining on Day 3 post‐ICH. *n* = 5–6. ***p < 0.001 versus sham; ^##^ *p* < 0.01 versus ICH + PBS. One‐way ANOVA followed by Tukey multiple comparison *post hoc* test was used for statistical analysis. (E) The number of activated microglia/macrophages was calculated by counting the active Iba‐1 positive cells on Day 3 post‐ICH. Each group includes a representative high‐magnification image positioned in the lower right corner. *n* = 5–6. ***p < 0.001 versus sham and ^###^ *p* < 0.001 versus ICH + PBS. One‐way ANOVA followed by Tukey multiple comparison *post hoc* test was used for statistical analysis.

Neurological and behavioral assessments on Day 3 post‐ICH showed marked enhancements in neurological function, forelimb strength, and motor coordination (Figure 5A). Histological analysis using Cresyl Violet/Luxol fast blue (CV/LFB) staining showed diminished lesion volume within the striatum following intranasal rIL‐10 administration on Day 3 post‐ICH (Figure 5B). FJB staining indicated a notable reduction in the density of degenerated neurons in the perihematomal region following intranasal rIL‐10 administration (Figure 5C). Perls' blue staining was employed to quantify iron deposition surrounding the hematoma, demonstrating a substantial reduction in iron accumulation in the perihematomal region on Day 3 post‐ICH with intranasal rIL‐10 treatment (Figure 5D). Moreover, assessment of microglia/macrophage activation within the perihematomal region revealed a significant reduction in the density of activated microglia/macrophages following intranasal rIL‐10 administration (Figure 5E). These encouraging results support the potential use of intranasal drug delivery therapy to treat ICH.

## 4 Discussion

In this study, we aimed to uncover the rIL‐10 distribution in the brain when administered intranasally. We labeled rIL‐10 with a fluorescent marker, CY5.5, and intranasally delivered it. Twelve hours later, we found the highest concentration of rIL‐10 around the hematoma in the brain. Significant amounts of rIL‐10 were also present in the cerebral cortex, striatum, and thalamus. Our results indicate that rIL‐10 primarily enters the brain through the olfactory nerve and then spreads through the brain along the spaces surrounding blood vessels. This study is the first to comprehensively investigate the distribution and timing of intranasally administered cytokines within the brain. Our results establish a robust basis for prospective clinical investigations of intranasal rIL‐10 therapy. It offers valuable insights into the underlying mechanisms of its therapeutic efficacy, instilling hope for potential breakthroughs in brain and spinal cord‐targeted treatments.

A recent study has revealed that the intranasal delivery of the anti‐inflammatory molecule IL‐10 can significantly enhance cognitive function in mice exhibiting depression‐like behaviors [14]. However, there is a lack of understanding regarding the optimal timing for intranasal rIL‐10 administration and its distribution within the brain after delivery. We conjugated rIL‐10 with the CY5.5 fluorescent tag to address this knowledge gap and employed advanced imaging techniques to monitor its spatial and temporal dynamics within the murine brain. Our findings indicate that a single administration of rIL‐10 remains detectable within the brain for 48 h before undergoing clearance, suggesting that the optimal therapeutic window for intranasal delivery falls within this time frame. Furthermore, imaging of brain sections demonstrated a peak concentration of rIL‐10 near the hematoma 12 h post intranasal administration, implying that a dosing interval of 12 h may yield optimal efficacy. These findings have significant implications for future research and the potential clinical applications of intranasal rIL‐10 administration for enhancing cerebral protection.

Additionally, our imaging procedure did not encompass the examination of the gastrointestinal tract due to potential interference from food and waste products. Notably, lung imaging revealed a notable presence of rIL‐10 4 h post intranasal administration, likely attributable to inhalation of the compound. Furthermore, intense endogenous fluorescence was observed in the liver of the control group, possibly stemming from red blood cells or their metabolic by‐products. Despite this, the liver was scrutinized further due to its pivotal role in drug metabolism.

Our investigations revealed that rIL‐10 reached the liver as the primary organ within 4 h, plausibly facilitated by the extensive capillary network in the nasal cavity. At 12 h post intranasal administration, rIL‐10 exhibited substantial accumulation in the liver, kidneys, and brain. By the 24 h mark, its distribution had extended to the brain and spinal cord, corroborating our observations from brain slice imaging.

Intranasal administration is a noninvasive approach for delivering drugs to the central nervous system, circumventing the blood–brain barrier. This method facilitates the transportation of various biological agents, including peptides [26], cells [27, 28], oligonucleotides [29], and viral vectors [30] to the central nervous system. Recent research on stroke patients indicated that 2 h after intranasal administration, the olfactory bulb shows the highest concentration of the drug BA‐PEG‐PLGA RNPs [31]. This observation suggests that the olfactory bulb may be a crucial entry point for drugs or compounds administered intranasally to access the brain. The olfactory and trigeminal nerves are the primary neural routes for intranasal drug delivery [32]. Among these, the olfactory neural pathway enables the transportation of drugs into neurons and their release in the olfactory bulb through the cytoplasmic and internalization pathways of olfactory sensory neurons [6]. Our identification of strong fluorescent signals in the olfactory nerve, in both the olfactory bulb and the piriform cortex, further supports the hypothesis that the drug is transported along this neural pathway.

The PVS within the brain encompasses the arterioles, capillaries, and venules, creating conduits for the movement of substances [33]. Recent research has demonstrated that the PVS can function as a pathway for drug distribution after intranasal delivery [34]. Our findings illustrate the substantial presence of fluorescent signals within the PVS of the olfactory bulb (granule cell layer) and cerebral cortex, indicating the widespread distribution of drugs along the PVS in the brain. However, the mechanism by which intranasally administered rIL‐10 is transported from nasal mucosa to the cerebral vasculature of the PVS remains a subject for future investigation. Our results suggest that intranasally administered rIL‐10 primarily reaches the brain through the perivascular space, with an additional route involving the olfactory nerve and pathway. These findings underscore the potential of the PVS as a promising route for drug delivery, inspiring us to explore this avenue further and potentially revolutionize the treatment of various brain disorders.

Recent research has revealed that intranasally administered substances such as insulin, IgG, and glucagon‐like peptide‐2 are present in the perineural spaces surrounding the trigeminal nerve. This finding suggests a potential role of the trigeminal nerve in the absorption and distribution of these substances following nasal application [35, 36, 37]. However, despite previous indications implicating the trigeminal nerve in intranasal drug delivery, recent studies have demonstrated that it may not be the primary conduit for translocating peptides and proteins from the nasal cavity to the brain [38]. The specific role of the trigeminal nerve in facilitating drug delivery to the brain remains the subject of ongoing investigation. Nevertheless, recent evidence suggests its potential involvement in mediating the transport of substances from the nasal cavity to the lower brain regions, including the brainstem [39]. Our findings corroborate this notion, as we observed notable levels of intranasally administered rIL‐10 in the brainstem.

One hour following intranasal administration, elevated levels of exogenous rIL‐10 were observed in the cerebral cortex, hypothalamus, and striatum. Subsequently, at the 12‐h mark, heightened rIL‐10 levels were detected in the cerebellum and brainstem. These observations suggest a gradual dissemination of rIL‐10 from initial target areas to more distant brain regions over time. The initially elevated rIL‐10 levels in the cortex, striatum, and thalamus may be attributed to the drug's transportation along the olfactory nerves. Notably, the cerebral cortex consistently exhibited the highest rIL‐10 levels, aligning with our prior findings [40], indicating that the cerebral cortex may serve as one of the primary targets for intranasal drug delivery. This modality holds promise for treating conditions associated with the cortex, such as Alzheimer's disease [41]. The prominent signal detected in the striatum supports the potential of exogenous rIL‐10 to enhance motor function after ICH [18]. High fluorescent signals in the thalamus also support the hypothesis that rIL‐10 alleviates pain and other affective states [42]. Intranasal administration of exogenous rIL‐10 resulted in the appearance of fluorescent signals in the hippocampus at 1 and 12 h post administration, suggesting that this mode of rIL‐10 delivery may enhance memory function [14, 40, 43]. Moreover, the noteworthy rIL‐10 levels in the brainstem support the plausibility of a pathway connecting the nasal cavity to the trigeminal nerve fibers in the brain [26].

Furthermore, in our preliminary experiments, we discovered that brain hemorrhage results in a significant amount of autofluorescence [23]. To guarantee the accuracy of our experiment, we established a control group to eliminate any background noise caused by autofluorescence. We also employed a unique technique known as SBB to minimize undesired autofluorescence in our images, enhancing the precision of our results.

The potential of intranasal drug delivery as a pharmacological approach is gaining recognition. In recent years, the intranasal administration of various drugs has shown excellent efficacy in treating neurological disorders. For example, oxytocin (OT) [42, 44, 45, 46, 47, 48, 49], insulin [44, 50, 51], and mesenchymal stromal cells [52] have demonstrated positive results. However, some studies have not shown efficacy, indicating the pressing need for further research [53, 54, 55, 56, 57].

This study examined how rIL‐10 is delivered to the brain following intranasal administration. Our findings show that rIL‐10 primarily enters the brain through the olfactory nerve and then spreads along the spaces surrounding blood vessels. These results have translational implications, providing strong evidence for using intranasal rIL‐10 in preclinical settings and highlighting its potential applications in treating different brain disorders.

## Author Contributions

S.W., Junmin W., C.J., X.F., and Jian W. conceived the study. S.W., Junmin W., X.Z., S.X., Q.P., Y.L., R.D., B.J., S.W., S.Z., S.H., Y.L., N.X., N.L., M.W., Junyang W., and X.C. performed the experiments and wrote the manuscript. S.W. and Junmin W. analyzed and reviewed the data. B.J., S.W., and S.Z. contributed to generating and analyzing in vivo imaging data. C.J., X.F., Junmin W., and Jian W. reviewed, revised, and edited the manuscript.

## Conflicts of Interest

The authors declare no conflicts of interest.

## Supporting information


**Figure S1.** Comparison of the fluorescence signals of CY5.5‐labeled recombinant IL‐10 on the hematoma and hematoma contralateral sides of intracerebral hemorrhage. *n* = 3. *p* > 0.05 versus hematoma contralateral; *F*_Interaction_ = 1.006, *F*_Row Factor_ = 3.866, *F*_Column Factor_ = 3.788; Two‐way ANOVA followed by Sidak multiple comparison *post hoc* test was used for statistical analysis.
**Figure S2.** Comparison of the fluorescence signal of CY5.5‐labeled recombinant interleukin‐10 (rIL‐10) in brain tissue affected by intracerebral hemorrhage before and after treatment with Sudan black B (SBB). After a single intranasal administration of CY5.5‐labeled recombinant interleukin‐10, fluorescence was detected near the hematoma in the same brain sections before and after SBB treatment. The white circles highlight areas showing significant differences in fluorescence signals before and after SBB treatment.
**Figure S3.** Statistics of fluorescence intensity in different brain regions on the hematoma side and the hematoma contralateral side of intracerebral hemorrhage 12 h after intranasal administration of recombinant IL‐10‐CY5.5. *n* = 3. *p* > 0.05 versus hematoma contralateral; *F*_Interaction_ = 0.04884, *F*_Row Factor_ = 5.460, *F*_Column Factor_ = 0.3195; Two‐way ANOVA followed by Sidak multiple comparison *post hoc* test was used for analysis.

## Data Availability

All data supporting this study's conclusions are available and can be obtained upon request from the corresponding authors. Raw data are provided with this paper.
