# Supplementary material for: Distribution of Intranasally Administered rIL‐10 Along the Olfactory Nerve and Perivascular Space After Intracerebral Hemorrhage
Source: CNS Neurosci Ther. 2025 Apr 16;31(4):e70372. doi: 10.1111/cns.70372 (PMC12000929; doi:10.1111/cns.70372)
Supplement: Supplementary file 1 — Figure S1. Comparison of the fluorescence signals of CY5.5‐labeled recombinant IL‐10 on the hematoma and hematoma contralateral sides of intracerebral hemorrhage. n = 3. p > 0.05 versus hematoma contralateral; F Interaction = 1.006, F Row Factor = 3.866, F Column Factor = 3.788; Two‐way ANOVA followed by Sidak multiple comparison post hoc test was used for statistical analysis. Figure S2. Comparison of the fluorescence signal of CY5.5‐labeled recombinant interleukin‐10 (rIL‐10) in brain tissue affected by intracerebral hemorrhage before and after treatment with Sudan black B (SBB). After a single intranasal administration of CY5.5‐labeled recombinant interleukin‐10, fluorescence was detected near the hematoma in the same brain sections before and after SBB treatment. The white circles highlight areas showing significant differences in fluorescence signals before and after SBB treatment. Figure S3. Statistics of fluorescence intensity in different brain regions on the hematoma side and the hematoma contralateral side of intracerebral hemorrhage 12 h after intranasal administration of recombinant IL‐10‐CY5.5. n = 3. p > 0.05 versus hematoma contralateral; F Interaction = 0.04884, F Row Factor = 5.460, F Column Factor = 0.3195; Two‐way ANOVA followed by Sidak multiple comparison post hoc test was used for analysis. [file CNS-31-e70372-s001.docx]

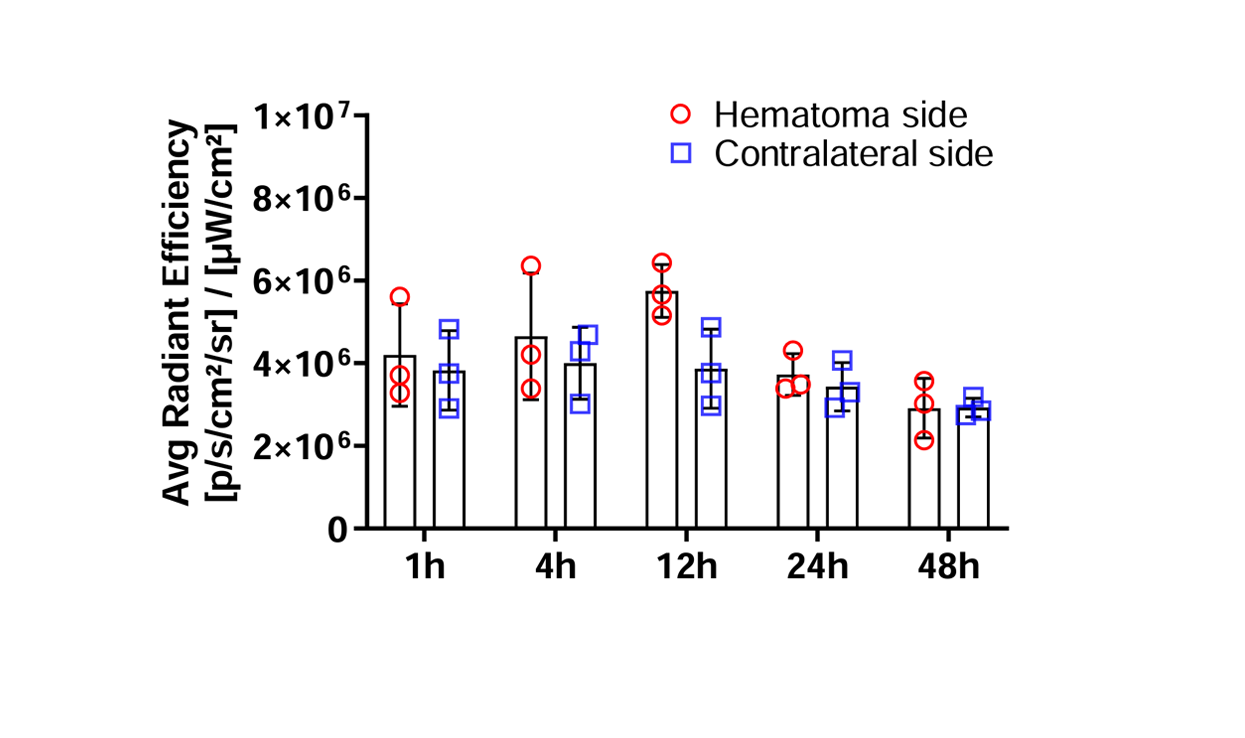


**Supplementary Figure 1.** Comparison of the fluorescence signals of CY5.5-labeled recombinant IL-10 on the hematoma and hematoma-contralateral sides of intracerebral hemorrhage. n=3. *P*＞0.05 versus hematoma contralateral; F_Interaction_=1.006, F_Row Factor_=3.866, F_Column Factor_=3.788; Two-way ANOVA followed by Sidak multiple comparison *post-hoc* test was used for statistical analysis.


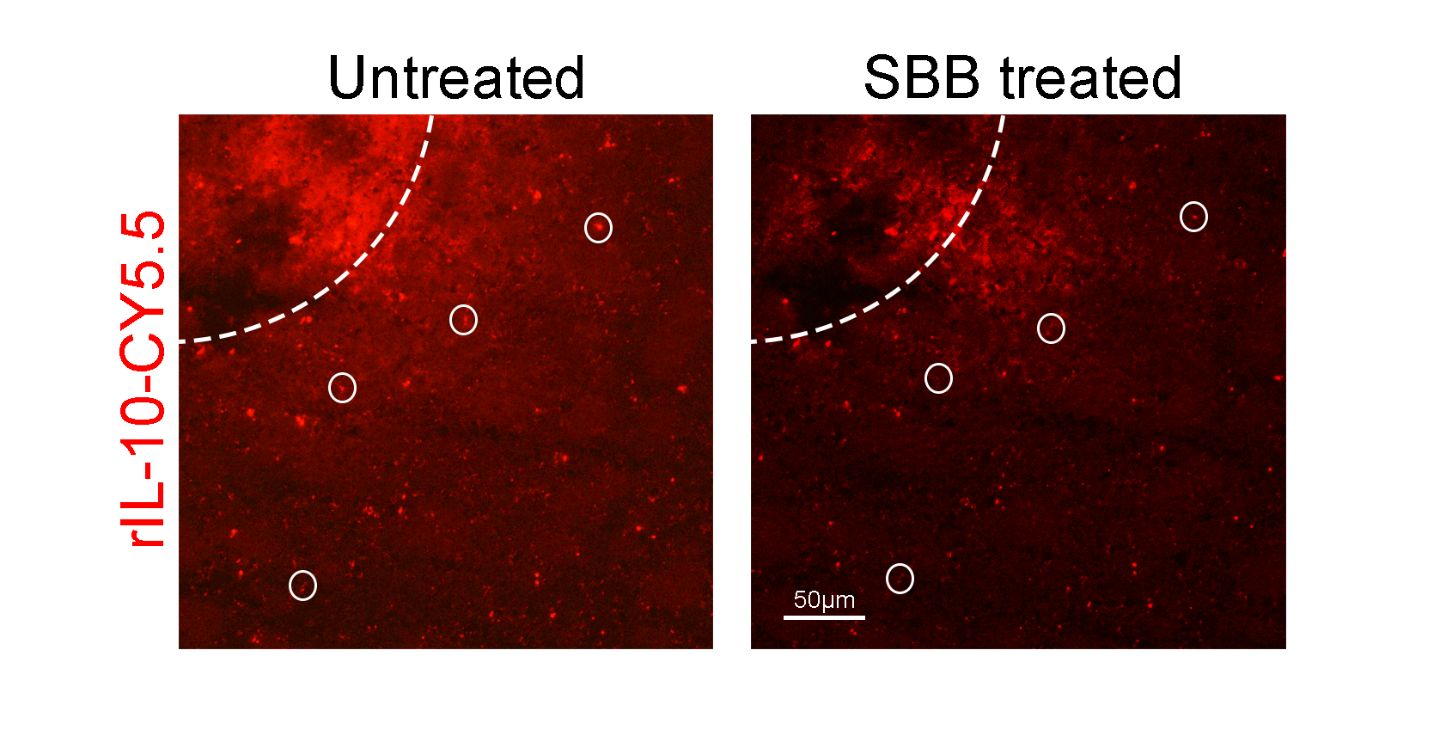


**Supplementary Figure 2. Comparison of the fluorescence signal of CY5.5-labeled recombinant interleukin-10 (rIL-10) in brain tissue affected by intracerebral hemorrhage before and after treatment with Sudan black B (SBB).**

After a single intranasal administration of CY5.5-labeled recombinant interleukin-10, fluorescence was detected near the hematoma in the same brain sections before and after SBB treatment. The white circles highlight areas showing significant differences in fluorescence signals before and after SBB treatment.


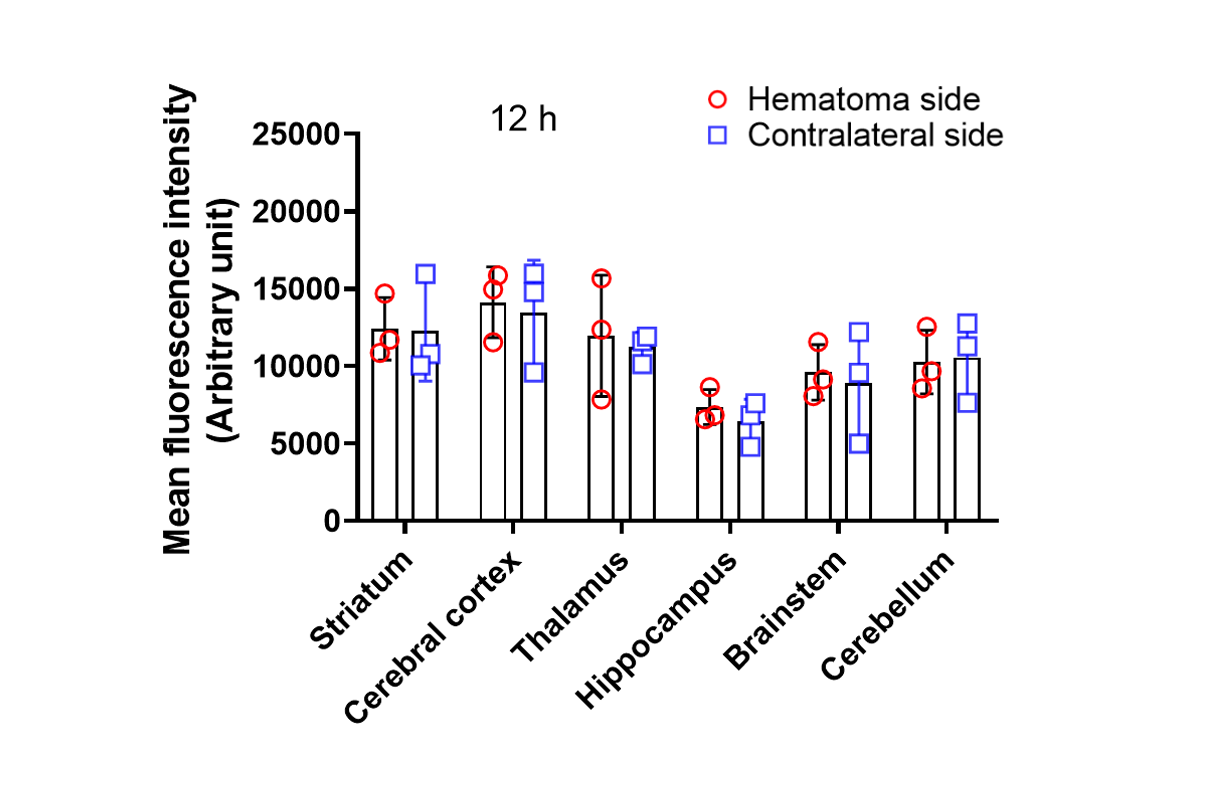


**Supplementary Figure 3. Statistics of fluorescence intensity in different brain regions on the hematoma side and the hematoma contralateral side of intracerebral hemorrhage 12 h after intranasal administration of recombinant IL-10-CY5.5. n=3.** *P*>0.05 versus hematoma contralateral; F_Interaction_**=**0.04884, F_Row Factor_=5.460, F_Column Factor_=0.3195; Two-way ANOVA followed by Sidak multiple comparison *post-hoc* test was used for analysis.
